# Comparison of the Degree of Gastric Mucosal Injury between Patients Who Are Receiving Dual Antiplatelet Therapy or Single Antiplatelet Therapy

**DOI:** 10.3390/diagnostics12102364

**Published:** 2022-09-29

**Authors:** Yuji Shimada, Mariko Hojo, Yuji Kita, Yuji Ikeda, Sho Sato, Ayato Murata, Shunsuke Sato, Kohei Matsumoto, Yoichi Akazawa, Tsutomu Takeda, Kumiko Ueda, Hiroya Ueyama, Tomoyoshi Shibuya, Takuya Genda, Akihito Nagahara

**Affiliations:** 1 Department of Gastroenterology and Hepatology, Juntendo University Shizuoka Hospital, Izunokuni 410-2295, Japan; 2 Department of Gastroenterology, Juntendo University School of Medicine, Tokyo 113-8421, Japan

**Keywords:** gastric mucosal injury, dual antiplatelet therapy, single antiplatelet therapy, low-dose aspirin, clopidogrel

## Abstract

Background: Patients taking low-dose aspirin have a higher incidence of gastroduodenal ulcers and higher risk of upper gastrointestinal bleeding than patients who do not. Thienopyridine antiplatelet agents may similarly cause bleeding gastroduodenal erosions and ulcers. The incidence of gastrointestinal bleeding is reported to be higher when these antithrombotic drugs are used in combination. Until now, most studies have focused on bleeding, and no study has compared the degree of gastric mucosal injury between patients receiving dual antiplatelet therapy (DAPT) and those receiving single antiplatelet therapy (SAPT) in real-world clinical practice. Aim: Our objective was to compare the degree of gastric mucosal injury in patients taking low-dose aspirin in combination with clopidogrel (one of the thienopyridine antiplatelet agents) with that of patients who were taking aspirin or clopidogrel as a single agent. Methods: Patients who were taking aspirin and/or clopidogrel and who underwent scheduled esophagogastroduodenoscopy between April 2015 and March 2020 were enrolled in this study. Endoscopic images were reviewed retrospectively, and the degree of gastric mucosal injury was assessed with the modified Lanza score (m-Lanza score). The m-Lanza score was compared between DAPT patients taking aspirin and clopidogrel and SAPT patients taking either aspirin alone or clopidogrel alone. Results: The m-Lanza scores of the DAPT group, the aspirin group, and the clopidogrel group were 1.67 ± 1.81 (mean ± standard deviation), 0.95 ± 1.61, and 0.72 ± 1.29, respectively. The m-Lanza score of the DAPT group tended to be higher than that of the aspirin group (*p* = 0.06) and was significantly higher than that of the clopidogrel group (*p* = 0.03). Conclusion: The degree of gastric mucosal injury in DAPT patients was significantly higher than that in patients using clopidogrel alone and tended to be higher than that in patients using aspirin alone in real-world clinical practice.

## 1. Introduction

Thienopyridine antiplatelet agents, which inhibit P2Y12 receptors and belong to the class of purine ADP-activated receptors, and low-dose aspirin are widely used to prevent stroke and cardiovascular events in patients with coronary artery disease [1,2]. However, low-dose aspirin users have a higher incidence of gastroduodenal ulcers and a higher risk of upper gastrointestinal bleeding than patients who do not take low-dose aspirin [3,4]. Thienopyridine antiplatelet agents may also cause erosions with bleeding and gastroduodenal ulcers [5,6]. Furthermore, it has been reported that dual antiplatelet therapy (DAPT) using these antithrombotic agents together is associated with a higher incidence of gastrointestinal bleeding than single antiplatelet therapy (SAPT) using one antithrombotic agent alone [7,8]. However, it has been recommended that stroke patients receive DAPT for a certain period of time to prevent recurrent stroke [9], and DAPT has become the standard treatment for prevention of stent thrombosis after percutaneous coronary intervention [10]. Therefore, DAPT is often administered after assessing the balance of risks and benefits.

Although many studies have evaluated gastrointestinal bleeding in patients receiving SAPT and those receiving DAPT with low-dose aspirin and a thienopyridine antiplatelet agent [11,12], few studies have evaluated how these antithrombotic agents actually injure the gastric mucosa [5,13,14], and no studies have compared the degree of gastric mucosal injury in patients receiving DAPT versus SAPT.

In this study, based on the findings of upper gastrointestinal endoscopy, we evaluated the difference in gastric mucosal injury between patients receiving DAPT with low-dose aspirin and clopidogrel (a thienopyridine antiplatelet agent) and patients receiving SAPT with low-dose aspirin or clopidogrel. 

## 2. Materials and Methods

### 2.1. Study Design

This study was a retrospective cross-sectional study. Data were extracted from medical records. The protocol used for this study was reviewed and approved by the Juntendo University Ethics Committee (S15-0427, date of approval 2 February 2016). The reporting of this study conforms to the Strengthening the Reporting of Observational Studies in Epidemiology statement [15].

### 2.2. Subjects

This study included patients who underwent scheduled esophagogastroduodenoscopy (EGD) in our hospital between April 2015 and March 2020. In conditions of overt bleeding such as bleeding gastric ulcer, the gastric mucosa cannot be adequately observed. Therefore, in the present study, we evaluated the degree of gastric mucosal injury in patients who had been scheduled to undergo endoscopy in the usual clinical practice. Exclusion criteria were patients who were taking neither low-dose aspirin nor clopidogrel at the time that EGD was performed; patients who were taking low-dose aspirin and/or clopidogrel along with a potassium-competitive acid blocker (PCAB), a proton pump inhibitor (PPI), a histamine 2 receptor antagonist, or another antithrombotic at the time when the endoscopy was performed; and patients who had a history of total gastrectomy.

### 2.3. Endoscopic Findings

Endoscopic images that had been taken by white light endoscopy using a video endoscopy system (GIF-H290, GIF-H290Z, GIF-XP290N, GIF-1200N, Olympus, Tokyo, Japan; EG-L600ZW7, EG-L580NW7, Fujifilm, Tokyo, Japan) were retrospectively viewed. The presence and degree of gastric mucosal injury were assessed based on the modified Lanza score (m-Lanza score). In this scoring system, the degree of gastric mucosal injury is determined as follows: 0, no evidence of erosions or hemorrhages; 1, no more than 2 erosions or hemorrhages confined to one anatomic area; 2, 3–5 erosions or mucosal hemorrhages confined to one area; 3, erosions or hemorrhages in 2 areas, or more than 5 erosions or mucosal hemorrhages in one anatomic area or 6–10 in the entire stomach; 4, erosions in 3 or more areas in the stomach or spread over the entire stomach in large numbers; and 5, gastric ulcer. Anatomic areas of the stomach were the prepyloric antrum, body, proximal antrum, and fundus. Ulcer was defined as a lesion >0.5 cm in diameter with a definite depression in the mucosal surface [16]. An m-Lanza score of zero was considered as no gastric mucosal injury, and an m-Lanza score of 3 or above, which indicates that erosions exist at multiple anatomic areas of the stomach, was considered as severe gastroduodenal mucosal injury in this study. The presence or absence of atrophy was diagnosed, and if atrophy was present, the degree of atrophy was evaluated according to the Kimura–Takemoto classification and classified into two types based on the location of the endoscopic atrophic border, which was endoscopically recognized by discriminating differences in the color and height of the gastric mucosa: closed type and open type [17].

The endoscopic images were judged by an endoscopist who was board-certified by the Japan Gastroenterological Endoscopy Society (YS) and who was blinded to which antiplatelet agent(s) was used by the patients.

### 2.4. Statistical Analysis

The patients were divided into three groups: those taking aspirin alone (aspirin group), those taking clopidogrel alone (clopidogrel group), and those taking both aspirin and clopidogrel (DAPT group). Continuous variables with normal distribution were expressed as means ± standard deviations. Baseline characteristics of each group were compared using chi-square test for categorical variables and analysis of variance for continuous variables. Regarding the m-Lanza score, the Kruskal–Wallis test was used to compare the m-Lanza score among the DAPT group, aspirin group, and clopidogrel group, and the Mann-Whitney U test was used for pairwise comparisons between the DAPT group and each of the aspirin or clopidogrel groups.

The chi-square test was used to determine if there was a significant difference in the degree of atrophy between cases without gastric mucosal damage and those with severe gastric mucosal damage.

*p*-Values of less than 0.05 were considered to be statistically significant. All analyses were undertaken using BellCurve for Excel version 3.21 (Social Survey Research Information Co., Ltd., Tokyo, Japan).

## 3. Results

**Figure 1:**
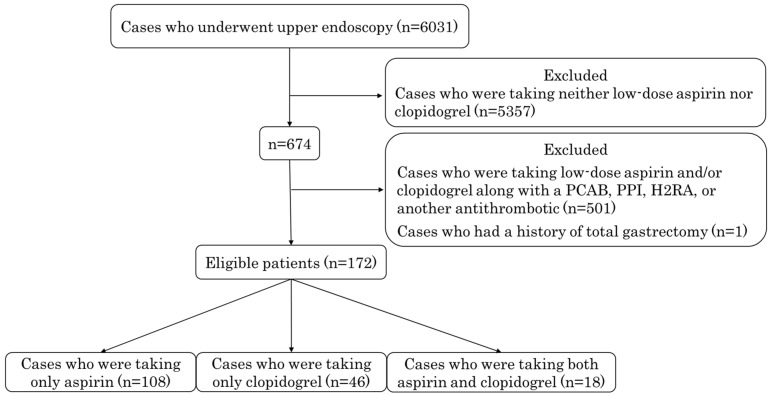
The flow chart of eligible patients. PCAB, potassium-competitive acid blocker; PPI, proton pump inhibitor; H2RA, histamine H2-receptor antagonist.

During the study period, 6031 patients underwent upper endoscopy, 5357 patients were excluded because they were taking neither low-dose aspirin nor clopidogrel, 501 patients were excluded because they were taking an excluded drug(s), and 1 patient was excluded because he had a history of total gastrectomy. A total of 172 patients were finally included in the analyses (Figure 1). The aspirin group included 108 patients, the clopidogrel group included 46 patients, and the DAPT group included 18 patients. The male-to-female ratio was 107:65, and their age at the time of endoscopy was 72.2 ± 11.3 years. The characteristics of the patients in each group, which were similar, are summarized in Table 1. There were no significant differences in gender or age among the three groups. There was also no significant difference in the percentages of patients who had no atrophy, closed-type atrophy, or open-type atrophy among the three groups.

**Figure 2:**
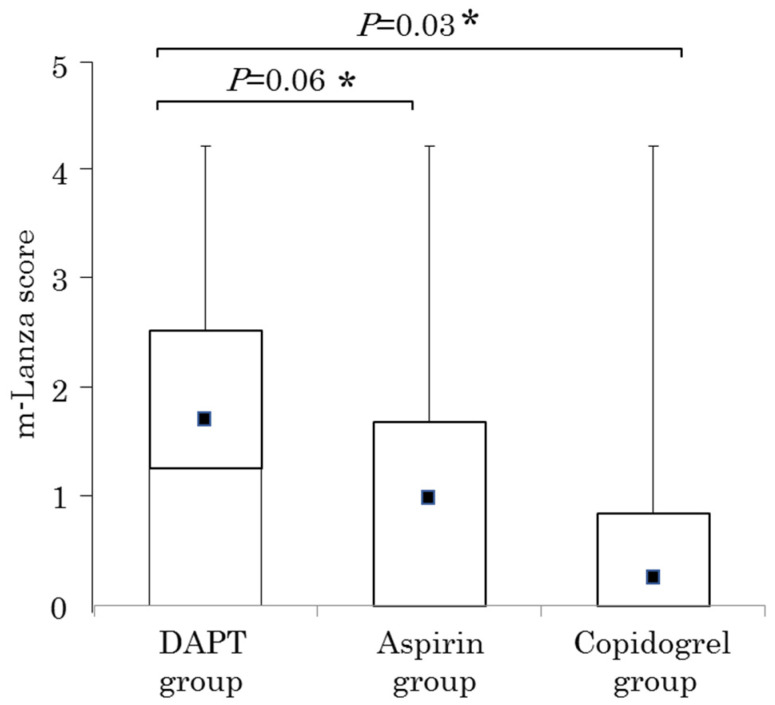
Box plots of the modified Lanza score (m-Lanza score) of the DAPT group, aspirin group, and clopidogrel group. ■ indicates the mean m-Lanza score. The m-Lanza score tended to be different among the DAPT group, aspirin group, and clopidogrel group (*p* = 0.09 by the Kruskal–Wallis test). * Between the DAPT group and each of the aspirin or clopidogrel group (*p* = 0.06 and *p* = 0.03, respectively, by the Mann–Whitney U test).

**Figure 3:**
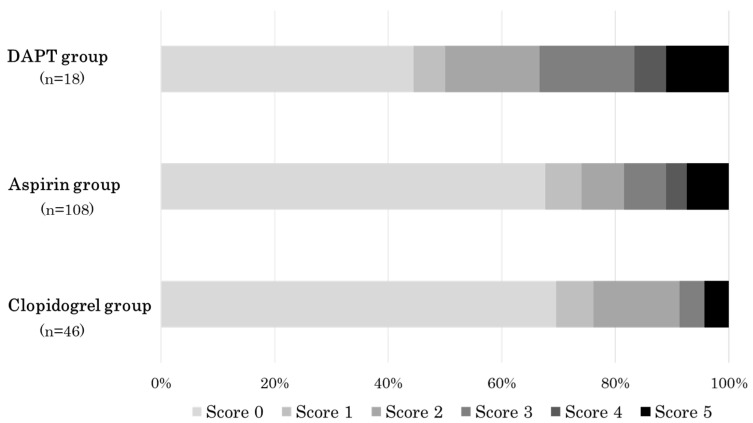
The proportions of patients with each modified Lanza score (m-Lanza score) in the DAPT group, aspirin group, and clopidogrel group. DAPT, dual antiplatelet therapy consisting of low-dose aspirin and clopidogrel.

Regarding the degree of gastric mucosal injury, the median (interquartile range) m-Lanza scores of the DAPT group, aspirin group, and clopidogrel group were 1.5 (0–3), 0 (0–2), and 0 (0–1), respectively, and the mean (± standard deviation) m-Lanza score was 1.67 ± 1.81, 0.95 ± 1.61, and 0.72 ± 1.29, respectively. The m-Lanza score tended to be different among the DAPT group, aspirin group, and clopidogrel group (*p* = 0.09 by the Kruskal–Wallis test). On pairwise comparisons, the m-Lanza score of the DAPT group tended to be higher than that of the aspirin group (*p* = 0.06 by the Mann–Whitney U test) and was significantly higher than that of the clopidogrel group (*p* = 0.03 by the Mann–Whitney U test) (Figure 2). Figure 3 shows the proportion of patients with each m-Lanza score in the DAPT group, aspirin group, and clopidogrel group.

Of the 172 patients, 30 had severe gastric mucosal injury. Of the 30 patients with severe gastric mucosal injury, 16 patients had no atrophy and closed-type atrophy (16/30, 53.3%). On the other hand, 113 of the 172 patients had no gastric mucosal injury; of the 113 patients with no gastric mucosal injury, 58 patients had no atrophy and closed-type atrophy (58/113, 51.3%). There was no significant difference in the degree of atrophy between those with severe gastric mucosal injury and those who did not have gastric mucosal injury.

## 4. Discussion

Previous papers investigating the association between low-dose aspirin and/or thienopyridine antiplatelet agents and gastric mucosal injury have focused on the presence or absence of bleeding [4,5], and how these agents cause gastric mucosal injury in real-world clinical practice has not been fully investigated. In conditions of overt bleeding such as bleeding gastric ulcer, the gastric mucosa cannot be adequately observed. Therefore, in the present study, we evaluated the degree of gastric mucosal injury in patients who had been scheduled to undergo endoscopy in the usual clinical practice. The results showed that the degree of gastric mucosal injury in DAPT patients tended to be higher than that in patients receiving aspirin monotherapy and was significantly higher than that in patients receiving clopidogrel monotherapy. It should be noted that DAPT was more prone to cause gastric mucosal injury than clopidogrel monotherapy. Aspirin monotherapy showed a similar trend as clopidogrel, but there was no significant difference in the degree of mucosal injury as assessed by the m-Lanza score between the aspirin and DAPT groups.

One possible reason for the lack of a significant difference in the m-Lanza score between the aspirin and DAPT groups was that the sample size was not large enough. However, it is also possible that the mucosal injury caused by aspirin is less severe than that caused by DAPT and more severe than that caused by clopidogrel.

Aspirin administered parenterally does not cause gastric mucosal injury despite suppression of prostaglandin E2 production in the gastric mucosa, suggesting the importance of topical toxicity on the occurrence of mucosal injury [18]. Aspirin is an acidic drug (pKa 3.5) and in the highly acidic stomach (pH 1–2) it exists in the molecular form and not in the ionic form. Therefore, it diffuses easily through the lipid bilayer into epithelial cells. Aspirin exists in the ionic form in epithelial cells and accumulates in the epithelium, causing direct mucosal injury. This further increases cell membrane permeability and causes mitochondrial oxidative phosphorylation damage [19,20,21,22]. On the other hand, clopidogrel has been reported to cause less gastric mucosal injury than aspirin [11]. Thirty-eight healthy volunteers were randomly assigned to receive either clopidogrel 75 mg or aspirin 325 mg. After taking the respective medication for 8 days, upper gastrointestinal endoscopy was performed, and the degree of gastric mucosal injury was evaluated by the Lanza score. The Lanza score of the clopidogrel group was significantly lower than that of the aspirin group [14]. However, clopidogrel inhibits angiogenesis and delays mucosal wound healing [23]. In a study in rats, Luo et al. [23] reported that clopidogrel administration delayed healing of gastric ulcers by inhibiting expression of vascular endothelial growth factor and inhibiting angiogenesis. Therefore, it can be hypothesized that aspirin directly causes gastric mucosal injury, and clopidogrel alone does not cause mucosal injury, but the delayed wound healing effect of clopidogrel causes the mucosal injury caused by aspirin to persist. The present results support this hypothesis. It has been reported that concomitant use of two antithrombotic drugs is associated with a high incidence of gastrointestinal bleeding [7]. The results of the present study demonstrated that gastric mucosal injury was more severe in DAPT patients than in patients who received clopidogrel alone, even in real-world clinical practice where antithrombotic agents are prescribed. Administration of a PPI or PCAB at the time of low-dose aspirin administration is only allowed in patients with a history of gastric ulcer or duodenal ulcer in the Japanese insurance system. PPIs and PCABs may not be prescribed for patients without a history of ulcers in Japan, even if they are taking aspirin. However, in patients receiving DAPT consisting of low-dose aspirin and a P2Y12 inhibitor such as clopidogrel, oral PPI or PCAB therapy may be necessary to prevent mucosal injury [24]. There is some suspicion that PPIs may influence the platelet aggregation effect of clopidogrel [25,26], but at this time it is believed that PPIs have no effect on the clopidogrel-induced inhibition of platelet aggregation [27,28,29].

Sogabe et al. reported that closed-type atrophy was associated with an increased incidence of severe gastroduodenal mucosal injury [30]. Therefore, it would be expected that the proportion of patients with closed-type atrophy would be higher among patients with severe gastric mucosal injury than among patients with no gastric mucosal injury. However, in the present results, there was no difference in the degree of atrophy between patients who had severe gastric mucosal injury and those who had no gastric mucosal injury. The reason for this is not clear but may be due to the fact that the present study examined only patients with gastric mucosal injury, while the study of Sogabe et al. examined patients with gastroduodenal mucosal injury, and that the percentage of patients with open-type atrophy was 47.1% in this study and 72.0% in the study of Sogabe et al.

The present study has several limitations. This study was a retrospective cross-sectional study performed at a single university hospital. Therefore, the number of cases was small. Gastric mucosal blood flow is important to maintain the integrity of gastric mucosa. Low mucosal blood flow predisposes to gastric mucosal injury, whereas high blood flow protects gastric mucosa against injurious agents and accelerates the healing of damaged mucosa [31,32,33,34,35]. It has been reported that blood flow also plays a protective role against mucosal injury in other organs of the gut such as the duodenum and colon [36,37]. Information on gastric mucosal blood flow is important. However, we could not obtain information about coexisting diseases and the general condition of the patients that might affect gastric mucosal blood flow, because it was not possible to obtain information beyond what was available in the medical records. We could also not obtain information about the duration of use of antithrombotic medications and *Helicobacter pylori* infection status, which are also factors that influence the development of gastric mucosal injury [38,39,40]. It is necessary to examine the effects of DAPT and SAPT on the gastric mucosa, taking into account the influence of these factors in the future.

## 5. Conclusions

DAPT patients had a higher m-Lanza score, indicating a higher degree of gastric mucosal injury than patients taking clopidogrel alone (*p* < 0.05) or aspirin alone (*p* = 0.06) in real-world clinical practice. It should be noted that when clopidogrel and aspirin are used together, gastric mucosal injury is more likely to occur than when each is used as a single agent.

## Figures and Tables

**Table 1:** Characteristics of the eligible patients. * Gastric mucosal atrophy was evaluated in the endoscopic images according to the Kimura–Takemoto classification, and was classified by degree into two grades of closed type and open type. DAPT, dual antiplatelet therapy; SD, standard deviation.

	Aspirin Group	Clopidogrel Group	DAPT Group	*P*
Cases (n)	108	46	18	
Gender [male/female (n)]	62/46	30/16	15/3	0.80
Age [mean ± SD (years)]	73.7 ± 12.1	69.6 ± 9.7	70.4 ± 8.9	0.81
Atrophy * [no atrophy/closed type/open type (n)]	37/22/49	16/10/20	4/2/12	0.53

## Data Availability

The data presented in this study are available on request from the corresponding author.
